# Incisal Interference Correction after Severe Extrusive Luxation Trauma during Orthodontic Treatment

**DOI:** 10.1155/2022/7181481

**Published:** 2022-11-15

**Authors:** M. Aarts, C. M. Suttorp

**Affiliations:** Section of Orthodontics and Craniofacial Biology, Department of Dentistry, Radboud University Medical Center, Nijmegen, Netherlands

## Abstract

This case report presents the treatment of a 12-year-old boy who suffered serious dental trauma, accompanied with buccal alveolar socket wall fractures, during orthodontic treatment. The maxillary right lateral incisor and canine were severely extruded and laterally luxated to the palatal side, resulting in an anterior crossbite and creating an incisal interference that prevented the jaws from closing in normal occlusion. During emergency treatment, the dentist attempted manual repositioning, but both severely extruded teeth were found to be immobile. One day after trauma, orthodontic repositioning was started using full fixed appliances with light 0.012″ nickel–titanium round wires, and this occlusal interference was corrected within 3 weeks. In the various internationally recognized treatment guidelines, the options for orthodontic repositioning for dental luxation trauma are only marginally described. Internationally accepted dental treatment guidelines may include immediate orthodontic repositioning after trauma as a treatment option in the management of dental extrusive and lateral luxation trauma.

## 1. Introduction

Many orthodontic treatments are performed in preadolescents; however, traumatic dental injuries are common during the preadolescent years [1]. Sooner or later, orthodontists and dentists will, therefore, see preadolescent patients who have suffered serious dental injuries. In the extrusive luxation [2] and lateral luxation [3], the tooth is axially out and non-axially displaced within the alveolar bone, respectively. Extrusively luxated teeth are also often luxated laterally to the lingual/palatal side, which can result in an anterior crossbite, leading to disruption of the occlusion [4]. The International Association of Dental Traumatology (IADT) recommends repositioning of laterally luxated and extruded permanent teeth, either manually or with forceps, shortly after trauma and stabilization with a flexible splint [5, 6]. However, alveolar bone deformations sometimes form a barrier to this tooth repositioning [7]. When treatment is delayed, blood clots are also found to be able to block the repositioning of luxated teeth [8]. In these situations, the orthodontist or dentist cannot immediately correct the occlusal interference, and direct orthodontic repositioning of the traumatically extruded teeth may then be considered [9]. However, starting orthodontic correction of extrusively or laterally luxated teeth shortly after trauma is not listed as a treatment option in the IADT's Dental Traumatology Guidelines [9], in the Dental Trauma Guide [5, 6], or in the guidelines of the European Society of Endodontology (ESE) [10]. Little has been published on immediate orthodontic correction of teeth after extrusive and lateral luxation trauma [11]. This case report aims to demonstrate the benefits of immediate orthodontic repositioning after extrusive and lateral luxation trauma. A selection from the available literature on this subject is also discussed.

## 2. Case Presentation

### 2.1. Diagnosis and Etiology

#### 2.1.1. Phase I

**Figure 1:**
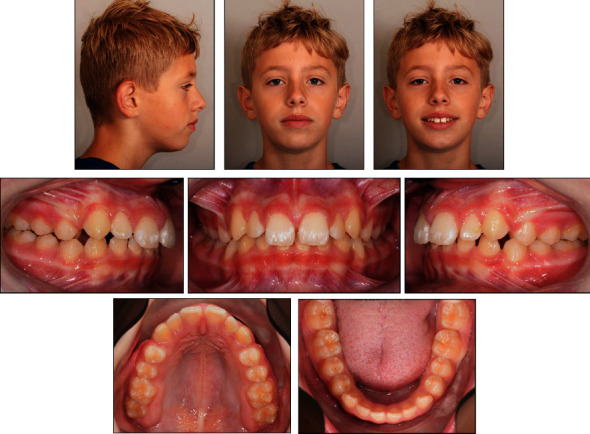
The patient's facial and intraoral photographs taken before treatment at the age of 12 years and one month.

At the age of 12 years and one month, the patient visited our clinic with complaints about the spaces between his maxillary and mandibular incisors, and requested orthodontic correction. The patient's profile appeared slightly convex with a hyperdivergent vertical skeletal pattern and a competent lip seal. An Angle Class I molar relationship with an increased overjet (6 mm) and overbite (5 mm) with a deep Curve of Spee was diagnosed. Minor spacing between the maxillary and mandibular incisors was found. The maxillary midline was approximately 1 mm off to the left side. The maxillary incisors and canines showed some enamel erosion (Figure 1).

**Figure 2:**
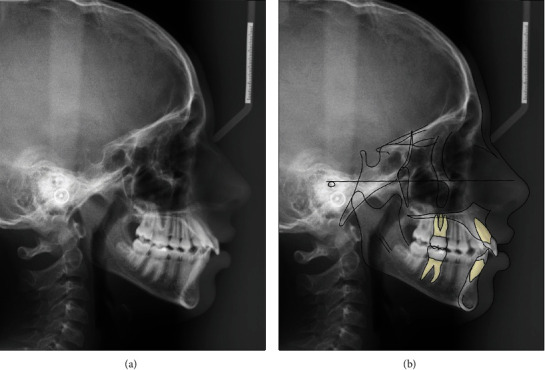
(a) Cephalometric radiograph (b) and cephalometric tracing before treatment.

**Figure 3:**
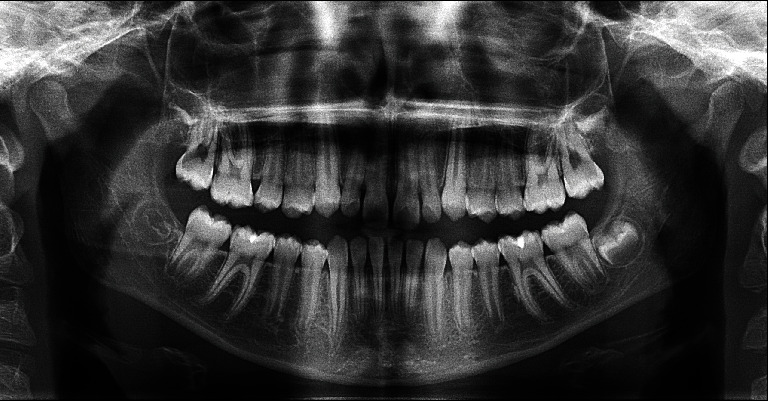
Panoramic radiograph before treatment.

The cephalometric analysis showed a retrognathic maxilla and mandible, a normal sagittal relationship between both jaws, a hyperdivergent vertical skeletal pattern, a retroclined palatal plane, and an anterior position of the lower lip (Figure 2; Table 1). The panoramic radiograph revealed four developing third molars (Figure 3).

**Figure 4:**
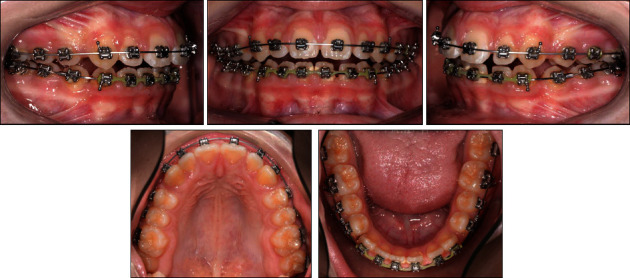
The patient's intraoral photographs taken after 8 months of orthodontic treatment.

The orthodontic treatment was started to correct the deep bite by leveling the deep Curve of Spee in the mandibular arch. A removable plate with an anterior bite plane was placed in the maxilla. In the mandibular arch, full fixed appliances were placed with a 0.018^″^ × 0.025″ slot in the incisor and canine regions, and 0.022^″^ × 0.025″ slot in the premolar and molar regions (In-Ovation R; Dentsply GAC, Islandia, NY, USA). Alignment and leveling were started with a 0.014″ nickel–titanium (Ni–Ti) round wire and completed within 6 months with, respectively, 0.018^″^ × 0.018″ Ni–Ti, 0.016^″^ × 0.022^″^ Ni–Ti, and 0.016^″^ × 0.022^″^ stainless steel (SS) wires (Dentsply GAC). Thereafter, removable plate wear was discontinued, and the maxillary dental arch was aligned within 4 months with the same type of fixed appliances as in the mandibular dental arch (Figure 4).

#### 2.1.2. Phase II

**Figure 5:**
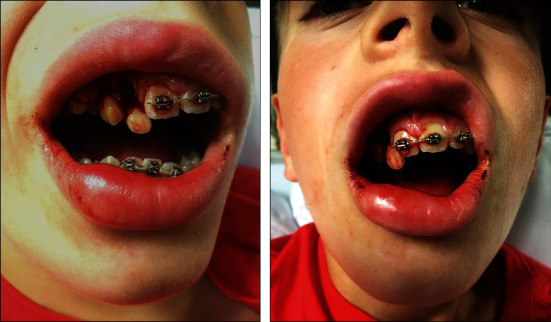
Intraoral pictures taken by the patient shortly after emergency dental treatment.

During the summer holidays, after 10 months of orthodontic treatment, the patient informed us by telephone that he had suffered a dental trauma. The patient had hit his front teeth with his own knee while doing a somersault on the trampoline at the campsite. Noting that several front teeth had been severely displaced, the patient immediately contacted the most nearby dentist for emergency treatment. The 0.016^″^ × 0.022^″^ SS wires in both the maxillary and mandibular dental arch were found to be heavily deformed. The dentist had first cut the SS wire in the maxillary dental arch and then removed the braces from the maxillary right lateral incisor, canine, and first premolar. Subsequently, the dentist had attempted to correct the displaced maxillary front teeth by manual repositioning, but these teeth were found to be immobile. The dentist undertook no further treatment. After that, the patient had taken intraoral photographs of the displaced teeth with his smartphone (Figure 5).

The same day the patient contacted our clinic for further treatment. During that phone call, we discussed the photographs of the dental trauma and advised the patient to visit our clinic as soon as possible. But the patient was not able to visit our clinic until the next morning.

**Figure 6:**
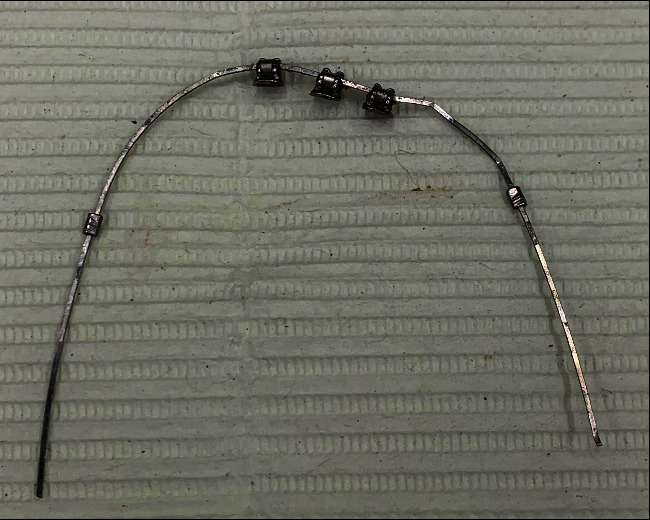
Photograph of the deformed 0.016^″^ × 0.022^″^ SS wire from the mandibular dental arch after dental trauma. The impact of the trauma had detached the brackets from the mandibular right central and lateral incisors and the mandibular left central incisor.

One day after the dental trauma, we saw the patient at our clinic together with the patient's home dentist. The upper and lower lips appeared diffusely swollen. The maxillary right lateral incisor and canine were severely extruded (about 5–6 mm). The maxillary left and right central incisors, the mandibular left and right central incisors, and the mandibular right lateral incisor and canine were also found to be extruded, but to a lesser extent (about 2–3 mm). All traumatically extruded teeth in the maxilla and mandible were laterally displaced to the palatal and lingual sides, respectively. The tooth displacement caused an anterior crossbite between the maxillary and mandibular right lateral incisors and canines, resulting in an incisal interference that prevented the jaws from closing in normal occlusion. The SS wire in the mandibular dental arch (Figure 6) and the still partially present SS wire in the maxillary dental arch were removed.

**Figure 7:**
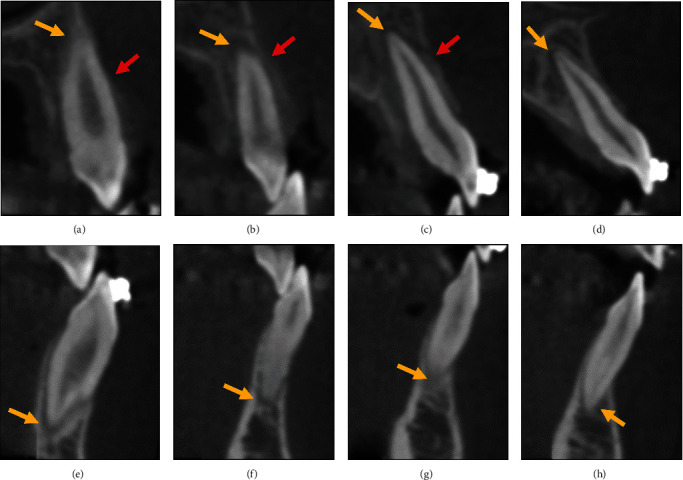
Dental CBCT images of the luxated teeth in the maxilla and mandible. Sagittal sections through the (a) maxillary right canine, (b) maxillary right lateral incisor, (c) maxillary right central incisor, (d) maxillary left central incisor, (e) mandibular right canine, (f) mandibular right lateral incisor, (g) mandibular right central incisor, and (h) mandibular left central incisor are shown. The red arrows indicate the location of a buccal alveolar socket wall fracture. The yellow arrows indicate the regions with an enlarged periodontal ligament space.

It was decided to take a dental cone-beam computed tomography (CBCT) image of the maxillary and mandibular volume (0.3 mm voxel size) to look for alveolar bone- and root-fractures. On the CBCT image, the buccal alveolar socket wall was fractured in the region of the maxillary right central and lateral incisors and the maxillary right canine. Furthermore, an enlarged periodontal ligament space was found at the apex of all extruded teeth (Figure 7). No fracture lines through the roots of the extruded teeth were found on the CBCT image.

We also attempted to reverse the premature contact by manual repositioning, but the maxillary and mandibular right lateral incisors and canines were again found to be immobile, as they were during the emergency treatment a day ago. Furthermore, the suspicion arose that some seriously displaced teeth would have lost their vitality. In joint consultation, it was therefore decided that in the coming months, the patient's home dentist will frequently examine the sensibility of all front teeth by ethyl chloride testing to monitor their vitality.

### 2.2. Treatment Alternatives

The patient requested immediate repositioning of the displaced teeth so that the jaws could close normally again. Immobile luxated teeth can be quickly replanted in their original position through the intentional replanting procedure [12]. This procedure consists of tooth extraction followed by irrigation, socket curettage, and almost immediate replanting of the tooth in its socket in its original position [13]. However, the recurrence of damage to the neurovascular bundle and periodontal ligament can lead to suboptimal treatment outcomes, as pulp necrosis and tooth ankylosis, respectively [2]. Therefore, the intentional replanting procedure was not selected.

The IADT's Dental Traumatology Guidelines recommend stabilizing the extruded and/or laterally luxated teeth for two weeks after repositioning and in the presence of alveolar bone fractures for four weeks with a passive flexible splint [5, 6]. These Dental Traumatology Guidelines further state that occlusal interferences are common in cases with displaced alveolar segments, but they do not recommend treatment options other than repositioning and passive splinting [5, 6]. However, since it was not possible to reposition the traumatically displaced teeth, along with the fractured buccal alveolar socket wall, it was chosen not to stabilize those teeth with a passive splint. Since the patient requested correction of the disturbed occlusion and the patient was still undergoing orthodontic treatment, immediate active orthodontic correction of the traumatically displaced teeth was preferred.

### 2.3. Treatment Progress

**Figure 8:**
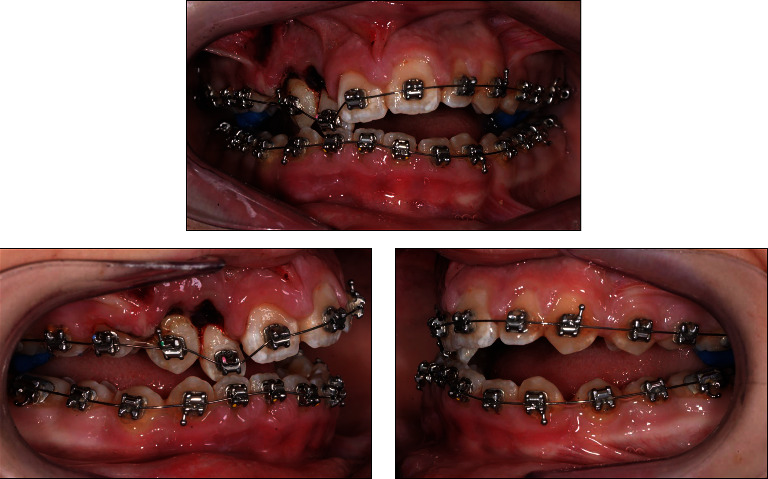
The patient's intraoral photographs taken directly after starting leveling and alignment with the 0.012″ Ni–Ti round wires, one day after the dental trauma.

At the patient's first visit to our clinic after the dental trauma, occlusal stops of light-cured band cement were immediately placed on the first maxillary molars to open the anterior bite. Brackets were placed on the maxillary right lateral incisor, canine, and premolar. Then, starting with light orthodontic forces in both dental arches, 0.012″ Ni–Ti round wires were placed (Figure 8).

**Figure 9:**
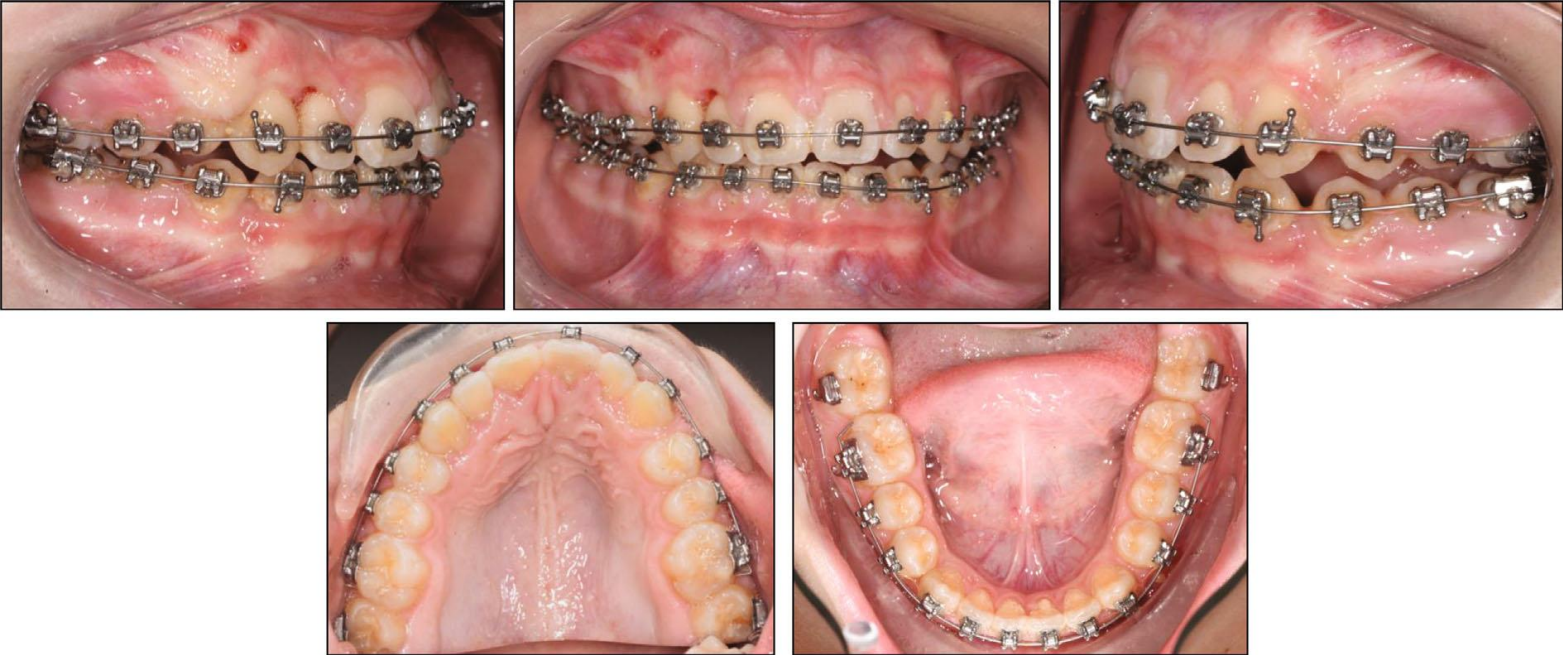
The patient's intraoral photographs taken after 3 weeks of leveling and alignment with the 0.012″ Ni–Ti round wires.

Three weeks later, orthodontic alignment had already largely corrected the crossbite between the maxillary and mandibular right lateral incisors and canines. The occlusal stops on the maxillary first molars were then removed (Figure 9). According to the patient, the crossbite was corrected shortly after placement of the fixed appliances, and the jaws were able to close in normal occlusion within a week. A fistula was noted on the buccal mucosa in the area between the apices of the maxillary right lateral incisor and canine.

**Figure 10:**
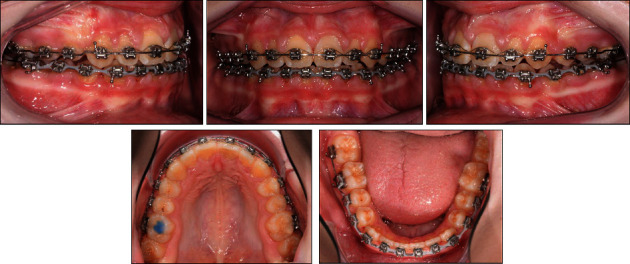
The patient's intraoral photographs taken during orthodontic finishing with 0.016^″^ × 0.022^″^ SS wires.

Within 4 months, both dental arches were then leveled and aligned sequentially with 0.012″, 0.018^″^ × 0.018″, and 0.018^″^ × 0.025^″^ Ni–Ti wires. A 0.016^″^ × 0.022″ SS wire was placed in both dental arches, and then Class II elastic traction (3.5 oz, 1/4″) was started to achieve optimal sagittal relationship between the front teeth (Figure 10).

**Figure 11:**
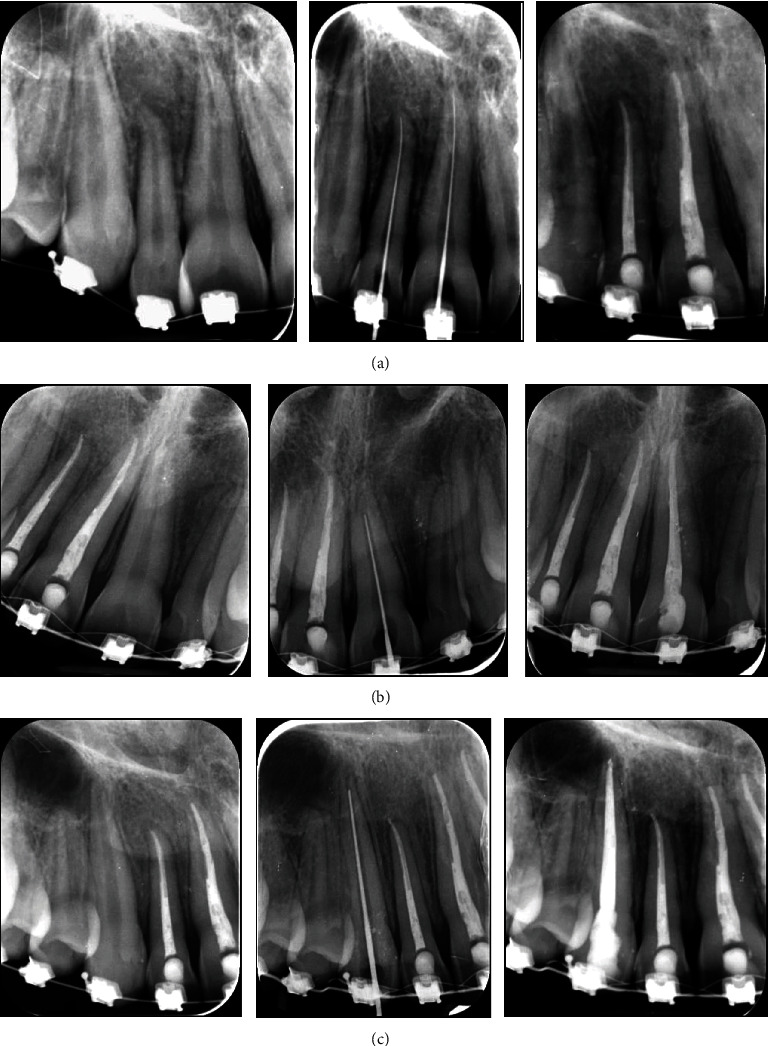
Periapical radiographs of the endodontic treatment of the (a) maxillary right central and lateral incisors, (b) maxillary left central incisor, and (c) maxillary right canine. The periapical radiographs (left) before endodontic treatment, (middle) during root canal working length measurement, and (right) after endodontic preparation and root canal and filling.

Ultimately, root canal treatments were performed by the dentist in the maxillary right central incisor, lateral incisor and canine, and the maxillary left central incisor (Figure 11).

Ten months after the dental trauma, the patient stated that he was satisfied with the treatment result and asked for debonding. The full fixed orthodontic appliances were removed, and a bonded canine-to-canine retainer (C–C bar) was placed in both dental arches. A removable (Hawley) retainer was placed to maintain the shape of the maxillary dental arch, and the patient was advised to wear this retainer overnight.

### 2.4. Treatment Results

**Figure 12:**
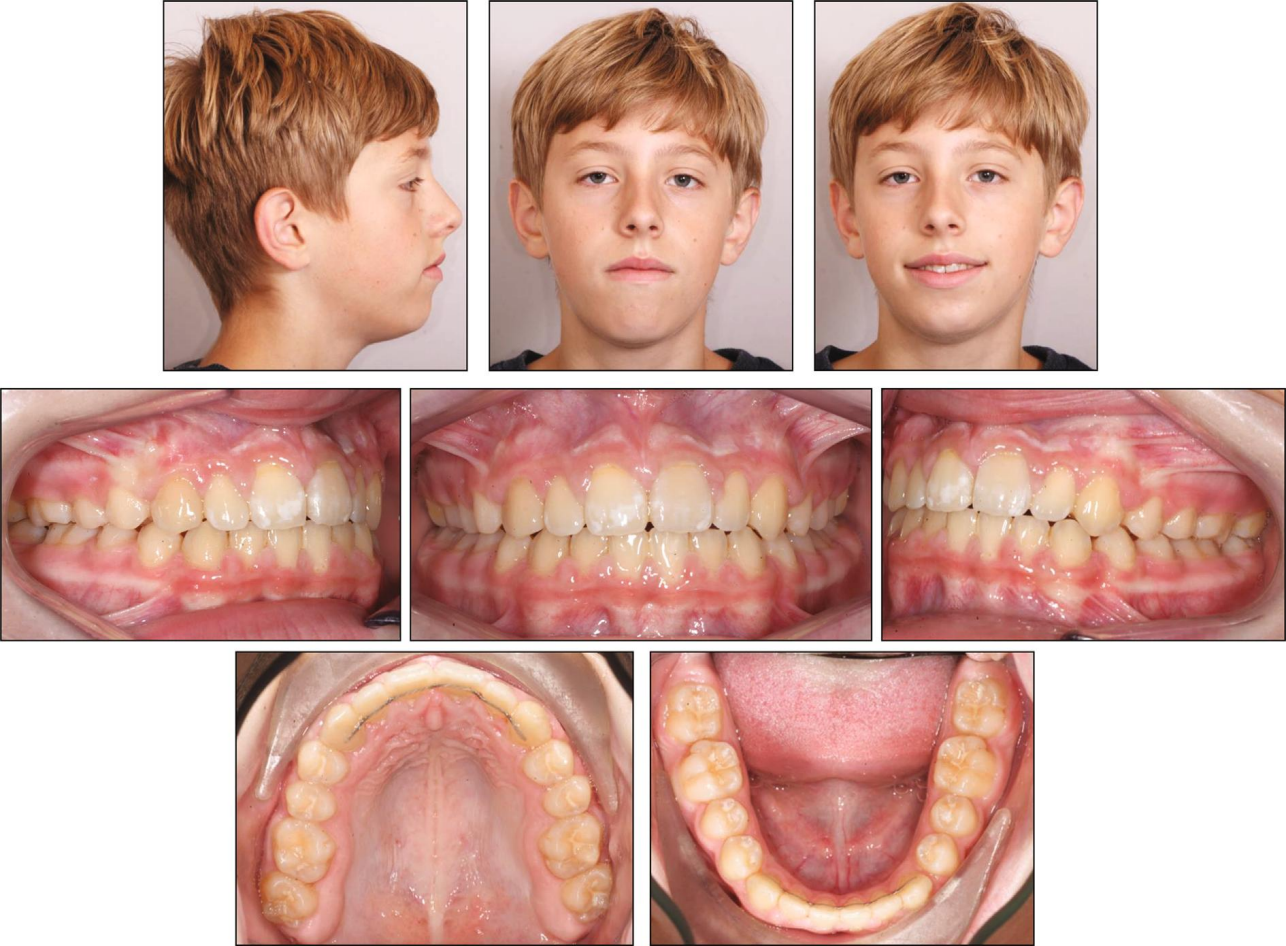
The patient's facial and intraoral photographs taken after treatment at the age of 13 years and ten months.

**Figure 13:**
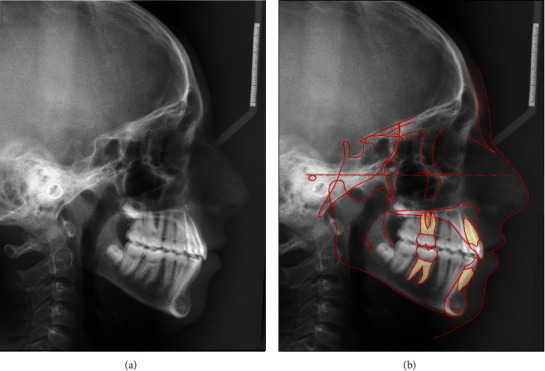
(a)Cephalometric radiograph and (b) cephalometric tracing after treatment.

**Figure 14:**
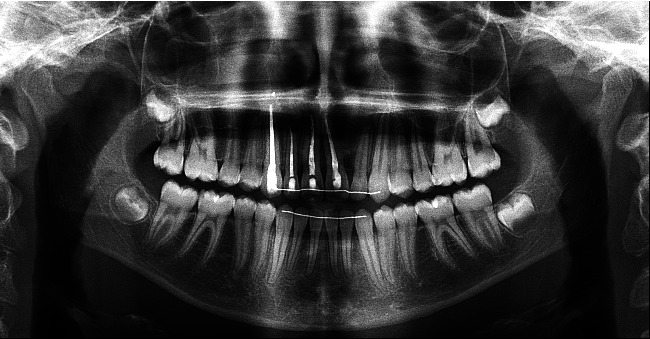
Panoramic radiograph after treatment.

At the day of debonding, facial and intraoral photographs (Figure 12), cephalometric radiograph (Figure 13(a)), and panoramic radiograph (Figure 14) were taken after removal of the orthodontic appliances. The extraoral photographs showed that the patient's slightly convex profile with a hyperdivergent vertical skeletal pattern was still present. The maxillary and mandibular left and right central incisors, and right lateral incisors and canines were all in an optimal position within the dental arch. The maxillary central diastema was closed. The small diastema (1 mm) behind both mandibular canines was still present, possibly due to the smaller width of the mandibular premolars compared to the width of the maxillary premolars. A Class I molar and canine occlusion with ideal overjet (2 mm) and overbite (2 mm) were achieved. More buccal root torque should have been applied to the maxillary right lateral incisor and canine.

The cephalometric analysis after treatment showed a retrognathic maxilla and mandible, but a normal sagittal jaw relationship. The hyperdivergent vertical skeletal pattern was maintained, and the vertical dentoalveolar complex had become hyperdivergent (Figure 13(b); Table 1). The endodontically treated maxillary right central and lateral incisors, canine, and maxillary left central incisor demonstrated no further pathology or complaints (Figure 14).

**Figure 15:**
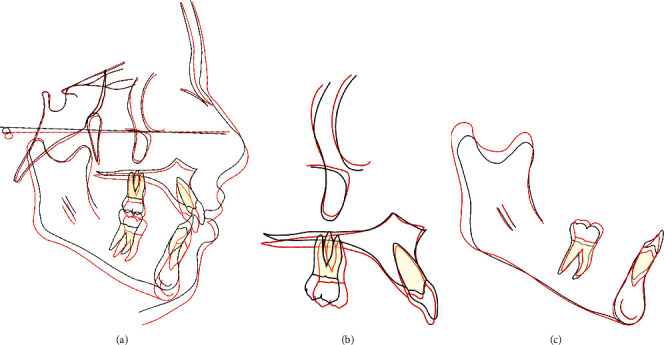
Cephalometric tracings before *(black lines)* and after treatment *(red lines)* were superimposed using Björk and Skieller's structural method. (a) General superimposition of the cephalometric tracings before and after treatment on the anterior cranial base. (b) Superimposition of the maxillary tracings before and after treatment on the anterior contour of the zygomatic process. (c) Superimposition of the mandibular tracings before and after treatment on the stable structures in the mandible.

Using Björk and Skieller's structural method [14, 15], the general superimposition of the cephalometric tracings before and after treatment on the anterior cranial base demonstrated an increase of vertical face height concurrent with expected growth. The hypodivergent skeletal pattern was maintained (Figure 15(a)). The superimposition of the maxillary tracings before and after treatment on the anterior contour of the zygomatic process showed a mesial displacement with a slight mesial tip of the first maxillary molars and a retroclination and slight extrusion of the maxillary incisors (Figure 15(b)). The superimposition of the mandibular tracings before and after treatment on the stable structures of the mandible (the protuberantia mentalis, the inner contour of the lower cortical plate of the symphysis, and the contours of the alveolar canals) demonstrated condylar growth and mandibular remodeling, along with extrusion and mesial displacement of the mandibular first molar and intrusion and retroclination of the mandibular incisors (Figure 15(c)).

**Figure 16:**
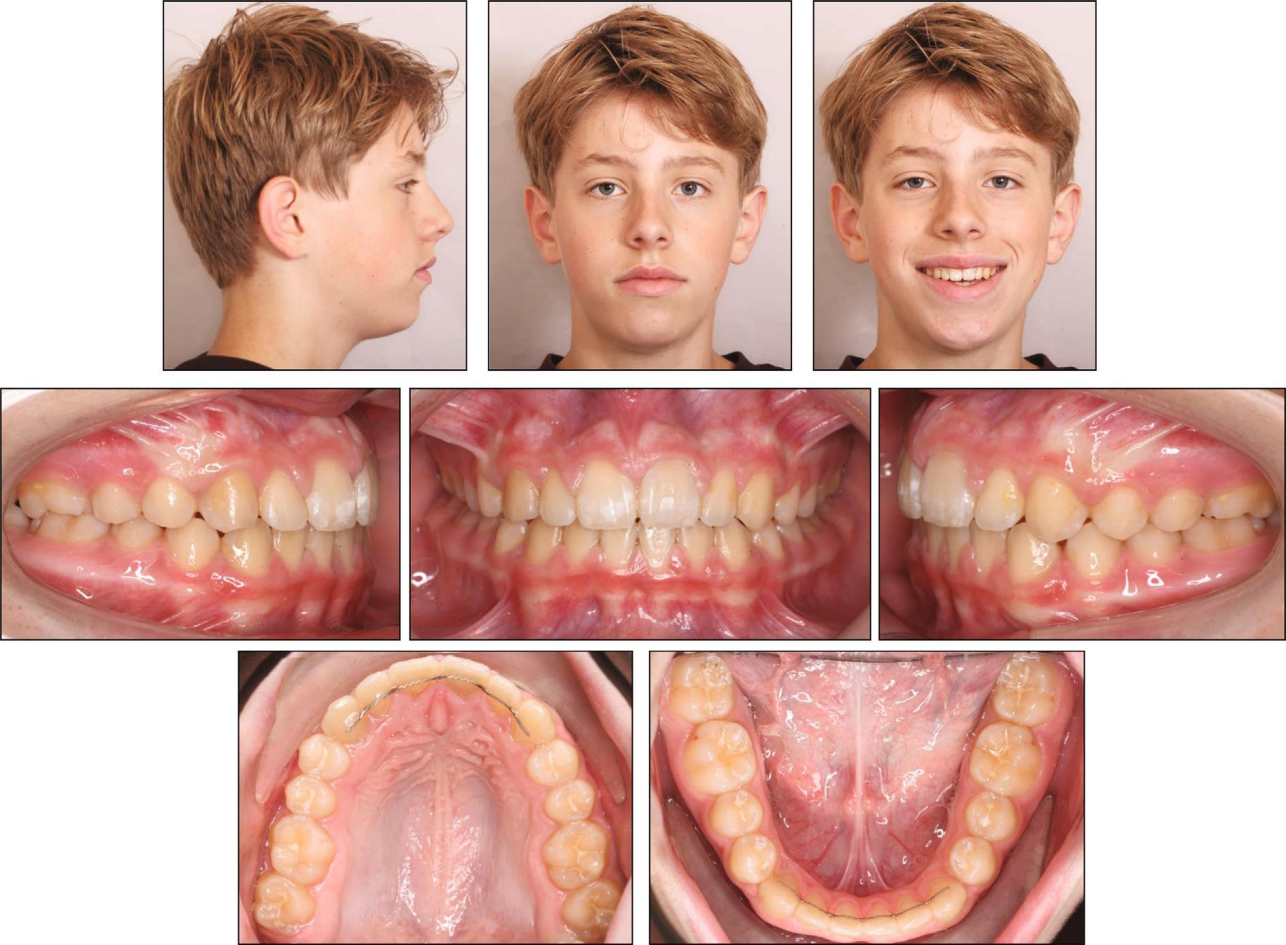
The patient's facial and intraoral photographs taken one year after treatment of the patient at the age of 14 years and ten months.

One year after treatment, occlusion and arch forms were still stable (Figure 16). The small diastema (1 mm) behind both mandibular canines had disappeared, probably due to the gradually improved interdigitation after debonding. Both bonded canine-to-canine retainers were still in place. The patient had worn the Hawley retainer overnight throughout the year. Since optimal interdigitation had been obtained, the patient was instructed to completely stop wearing the Hawley retainer. The patient reported that the treatment result was still satisfactory.

## 3. Discussion

In the Dental Trauma Guide [16], and in the dental trauma guidelines of the IADT [17] and the ESE [10], the use of orthodontic forces is only recommended for active extrusion of traumatically intruded teeth. In guidelines for the management of traumatic dental injuries of the American Association of Endodontists', the use of orthodontic forces is only recommended in cases with crown/root fracture trauma to extrude the apical root fragment to expose the margins prior to permanent restorations [18]. These guidelines do not mention the use of orthodontic forces in the treatment of other types of dental luxation trauma, such as extrusive and lateral luxation trauma [9]. Orthodontic repositioning is mentioned only marginally in treatment guidelines for dental luxation trauma [9]. Several dental trauma guidelines even advise to wait at least 6 months with the orthodontic correction of luxated teeth [19]. In contrast, the recent guidelines for the orthodontic management of the traumatized tooth of Sandler et al. do describe the use of orthodontic forces in extrusive and lateral luxation traumas [20]. Years ago, other practitioners already proposed the use of orthodontic forces immediately after extrusive and lateral luxation traumas, but the amount of data are limited [9]. Evidence that luxated teeth can be successfully repositioned by light orthodontic forces, using 0.012 or 0.014″ Ni–Ti round wires, immediately after trauma, is based only on clinical experience and some case reports [19, 21, 22]. However, it has not been scientifically proven that orthodontic repositioning immediately after extrusive and lateral luxation trauma is less successful than manual repositioning or forceps repositioning.

The purpose of splinting is to stabilize the teeth after trauma to promote periodontium regeneration [23]. It has been stated in the literature that the application and removal of splints after trauma should be easy and quick to perform without causing additional irritation to the surrounding tissues [23]. It can be stated that splints consisting of orthodontic braces and flexible orthodontic wires meet these criteria and can be used as both active and passive splints.

Since the patient already had some degree of enamel erosion in the incisors and canines in the maxilla, prevention of a further increase is desirable. The use of hydroxyapatite-based toothpaste has been shown to be effective in preventing enamel erosion [24], and its use may be considered for this patient.

In this case, the patient was not seen in our orthodontic clinic until a day after the trauma, which delayed the treatment. That the maxillary and mandibular right lateral incisors and canines were not manually repositionable can be explained by this delay in treatment. In other reports of orthodontic repositioning, treatment was also often delayed and usually occurred days or weeks after the trauma [9]. It has been reported that delaying treatment can lead to the formation of blood clots, which can block repositioning of the luxated teeth [8]. However, in a number of cases, orthodontic repositioning has been shown to be a treatment alternative when manual tooth repositioning cannot be performed [7, 9].

In this case, the patient reported that tooth repositioning began shortly after placement of the 0.012″ Ni–Ti round wire and that the crossbite was corrected within a week. Others found that by using fixed orthodontic appliances with light 0.012″ Ni–Ti round wires, laterally luxated teeth can be moved orthodontically after trauma, and immediate repositioning can even be completed in about 3–14 days [4, 25]. Our finding that orthodontic repositioning of luxated teeth can be started as early as day 1 and completed within days to weeks is consistent with the findings of others.

A few articles have described that laterally luxated or extruded teeth may have moved palatally, leading to an anterior crossbite with occlusal interference [4, 20, 26]. Owtad et al. placed posterior occlusal stops for assistance of the orthodontic correction in a case of trauma-induced anterior crossbite [19]. To correct the incisal interference in this case, we also placed occlusal stops, consisting of light-cured band cement, to get the palatally displaced maxillary right incisors and canine out of crossbite.

Radiologically, after extrusive luxation trauma, a tooth may show a larger periodontal ligament space [27], as well as also observed on the CBCT images in this case. Extrusive luxation trauma can cause periodontal ligament disruption, and damage mainly occurs to the intercellular structures [28]. In contrast, in lateral luxation trauma, the periodontal ligament is partially compressed and partially disrupted [28]. In the compressed areas of the periodontal ligament, the cells and collagen fibers are crushed [28]. Healing of the periodontal ligament after luxation injury involves restoration of the collagen fibers, as well as revascularization and reinnervation [28]. Periodontal ligament healing can take approximately two weeks if no further inflammatory stimulus is present [29]. Extensive periodontal ligament necrosis and damage to the root cementum may promote the repopulation of the damaged root areas with bone-producing cells, which may result in direct attaching of the bone with the root surface, called tooth ankylosis [30]. The absence of the periodontal ligament between the root cementum and the alveolar bone prevents ankylosed teeth from moving under orthodontic forces [30, 31]. It has been suggested that manual repositioning of luxated teeth can create large forces on the tooth, causing additional trauma to the periodontal ligament [32]. Therefore, it can be argued that orthodontic repositioning of luxated teeth using light orthodontic forces is preferable to manual repositioning with higher forces to prevent ankylosis. However, no studies have yet been conducted on the difference in periodontal damage resulting from orthodontic repositioning and manual/forceps repositioning in extrusive and laterally luxated teeth.

## 4. Conclusion

This case report has shown that immediate orthodontic repositioning of extruded or laterally luxated teeth can be a treatment option when manual repositioning of luxated teeth is not possible and/or the resulting incisal interference prevents the jaws from closing in normal occlusion. Internationally accepted dental treatment guidelines may include immediate orthodontic repositioning after trauma as a potential treatment option in the management of tooth extrusive and lateral luxation trauma.

## Figures and Tables

**Table 1:** Cephalometric measurements before and after treatment.

Measurements	Norm	Before treatment	After treatment
Skeletal			
SNA (°)	81.2 ± 3.3	**76.0**	**76.9**
SNB (°)	77.3 ± 2.7	**72.5**	**73.6**
ANB (°)	3.9 ± 2.1	3.4	3.3
Wits (mm)	—	2.3	−2.6
NSL/ML (°)	33.8 ± 4.9	**41.1**	**41.7**
NL/ML (°)	27.3 ± 4.9	29.8	**32.1**
NSL/NL (°)	6.5 ± 3.0	**11.3**	9.6
Dental			
ILs/NL (°)	110.5 ± 5.5	114.2	106.8
Is/APog (mm)	6.4 ± 2.3	8.5	5.6
Interincisal angle (°)	127.1 ± 9.7	126.4	131.4
ILi/ML (°)	95.4 ± 5.8	89.6	89.8
Ii/Apog (mm)	1.9 ± 2.3	3.1	2.8
Soft tissue			
Nasolabial angle (°)	110.8 ± 9.1	112.1	119.1
Ls to Sn–Pog' (mm)	3.7 ± 1.7	4.0	2.8
Li to Sn–Pog' (mm)	3.1 ± 1.8	4.9	3.2
Li to E-line (mm)	−2.1 ± 1.5	**2.0**	−0.6

Acronyms/full names: SNA = anteroposterior position maxilla; SNB = anteroposterior position mandible; ANB = sagittal jaw relationship; NSL/ML = (nasion–sella line/mandibular line) vertical skeletal pattern; NL/ML = (nasal line/mandibular line) vertical dentoalveolar complex; NSL/NL = (nasion–sella line/nasal line) inclination of the palatal plane; ILs/NL = (maxillary incisor inclination angle to nasal line) inclination of the maxillary incisors; Is/APog (maxillary incisor to A-pogonion line) anteroposterior position maxillary incisors; ILi/ML = (mandibular incisor inclination angle to mandibular line) inclination of the mandibular incisors; (Ii/Apog) = (mandibular incisor to A-pogonion line) anteroposterior position mandibular incisors; Ls to Sn-Pog' = (midpoint of the vermilion border of the upper lip to the subnasale-pogonion' line) anteroposterior position of the upper lip; Li to Sn-Pog' = (midpoint of the vermilion border of the lower lip to the subnasale–pogonion' line) anteroposterior position of the lower lip; Li to E-line = (midpoint of the vermilion border of the lower lip to the esthetic line) anteroposterior position of the lower lip. The cephalometric values beyond one standard deviation (SD) are shown in bold.

## Data Availability

Data supporting this research article are available from the corresponding author or first author on reasonable request.
